# Determination of the efficacy of EVICEL™ on blood loss in orthopaedic surgery after total knee replacement: study protocol for a randomised controlled trial

**DOI:** 10.1186/s13063-015-0822-y

**Published:** 2015-07-11

**Authors:** S. Budde, Y. Noll, V. Zieglschmid, C. Schroeder, A. Koch, H. Windhagen

**Affiliations:** Department of Orthopaedic Surgery, Hannover Medical School, Anna-von-Borries-Str. 1-7, 30625 Hannover, Germany; Hannover Clinical Trial Center GmbH, Carl-Neuberg-Str. 1, 30625 Hannover, Germany; Institute of Clinical Pharmacology, Hannover Medical School, Carl-Neuberg-Str. 1, 30625 Hannover, Germany; Institute of Biometry, Hannover Medical School, Carl-Neuberg-Str. 1, 30625 Hannover, Germany

**Keywords:** fibrin sealant, EVICEL, total knee arthroplasty, total knee replacement, transfusion rate, haemoglobin loss, blood loss, range of motion

## Abstract

**Background:**

After total knee replacement, overall blood loss is often underestimated, although it exceeds the visible blood loss caused by bleeding into the tissues or into the joint. The use of fibrin sealants during surgery has been suggested to reduce perioperative blood loss and transfusion rates and may be beneficial for patient recovery and the postoperative function of the joint.

**Methods/Design:**

This will be a single-centre, single-blinded, randomised controlled trial with a parallel design, for which 68 patients undergoing total knee replacement will be recruited and followed up at 3, 6 and 12 months; 34 will be control patients who will receive the standard orthopaedic surgery treatment (electrocoagulation), and the other 34 will receive the same treatment plus 5 ml EVICEL™ applied during surgery and used according to the manufacturer’s instructions. The primary objective is to test the null hypothesis that the effect of EVICEL™ for controlling haemostasis and reducing postoperative blood loss in patients undergoing total knee replacement is not superior to the use of electrocoagulation alone. The secondary objective is to show that EVICEL™ reduces the need for transfusion, increases range of motion, improves clinical outcome and wound healing, and reduces the need for analgesics. The tertiary objective is to show that EVICEL™ reduces the costs of total knee replacement treatment.

**Discussion:**

So far, studies on the effect of fibrin sealants in total knee replacement have delivered inconsistent and ambivalent results, indicating that there is still a need for high-evidence studies as proposed in the presented study protocol.

**Trial registration:**

German registration number DRKS00007564; date of registration: 26 November 2014.

## Background

Implementing a new technology means using a new device in existing procedures with the global target of improving health care, thereby leading to substantial improvements in patient outcomes.

In orthopaedic surgery, especially following total hip and total knee arthroplasty, the overall blood loss is often underestimated as it exceeds the visible blood loss due to bleeding into the tissues or into the joint [1]. A large loss of blood means stress to the cardiovascular system and retards the patient’s recovery process [2]. Some patients undergoing total knee replacement (TKR) require the transfusion of allogeneic blood products in order to avoid cardiovascular complications. In addition, a postoperative haematoma may lead to an impairment of knee range of motion (ROM) [2].

Since 1972, the supportive use of fibrin sealants in selected surgical procedures has become current practice to control haemostasis and to reduce blood loss after surgery. However, the use of fibrin sealants in orthopaedic knee and hip surgery, two procedures often associated with a considerable amount of postoperative blood loss, is not considered standard.

EVICEL™ is a fibrin sealant indicated for use as a supportive treatment in surgery for the improvement of haemostasis where standard surgical techniques are insufficient. Bearing in mind that a new orthopaedic surgery guideline was published recently recommending that acetylsalicylic acid (aspirin) or clopidogrel regimens in patients undergoing orthopaedic surgery not be reduced or interrupted [3], the use of EVICEL™ in daily clinical practice might contribute to a reduction of blood loss, especially in these patients. As a consequence the use of fibrin sealants might improve healing and reduce the impaired ROM, leading to less use of analgesics after surgery, shorter hospital stays and reduced total costs of TKR treatment.

## Methods/Design

### Study design

This study will be a single-centre, parallel-design, randomised controlled trial (RCT) in which 68 patients undergoing TKR will be recruited according to the inclusion/exclusion criteria for RCTs, and treated within a 12-month period at the Department of Orthopaedic Surgery. It is registered in the German registry under number DRKS00007564. Ethical approval has been obtained from the Institutional Review Board of the Hannover Medical School under process number ‘6170 M mono’. The study will be conducted in accordance with the Helsinki Declaration.

Thirty-four (34) control patients will receive the standard orthopaedic surgery treatment (electrocoagulation). Another 34 will receive the same treatment as control patients plus 5 ml EVICEL™ applied during surgery and used according to the manufacturer’s instructions.

The main trial period is the stay in hospital for the surgery and postoperative surveillance. Follow-up examinations of all patients will be conducted 3, 6 and 12 months after surgery. With a recruitment period of 12 months and a follow-up period of 12 months, the total length of the study period is calculated to be approximately 24 months.

The study will be terminated when the necessary 34 patients per group have completed the study. Additionally, the sponsor has the right to terminate the study at any time for reasonable medical or administrative reasons. Also, the principal investigator can decide to terminate the study at any time for reasonable medical or administrative reasons.

The results manuscript will follow the advice from the CONSORT guide and its extension to cluster trials.

### Trial objectives

This randomised controlled study has three main objectives. The primary objective is to test the null hypothesis that the effect of EVICEL™ on controlling haemostasis and reducing postoperative blood loss in patients undergoing TKR is no different than with the use of standard orthopaedic surgery. The secondary objective is to show that EVICEL™ reduces the need for transfusion and increases ROM when measured 7 days after surgery and in the long term, improves wound healing and reduces the need for analgesics. In addition, two clinical outcome scores will be assessed.

The tertiary objective is to determine the influence of EVICEL™ use on the overall cost of TKR treatment.

### Primary and secondary study endpoints

The primary endpoint of this study is postoperative blood loss after TKR, measured by the difference in Hb levels at baseline (recorded in the 3 days prior to randomisation) with respect to the detected minimum in the first 7 days postoperatively and compared between the study group and the control group. During this period, blood samples will be taken regularly according to the in-house protocol, and results will be compared with control values derived from patients treated under the same conditions but without the use of EVICEL™. During the operation, all factors that affect the postoperative level of Hb will be documented.

Secondary endpoints are the need for at least one allogeneic blood transfusion or one autologous transfusion, the ROM of the operated joint, the postoperative use of analgesics, time until wound healing, clinical outcome scores, length of hospital stay, and the overall cost of the treatment.

A follow-up measurement of ROM will be conducted 3, 6 and 12 months after surgery. The ROM of the operated joint in the study and the control group will be measured using the angle of maximum flexion. Factors that might influence the ROM (for example, the use of a peripheral nerve block) will be documented and might lead to exclusion from the secondary endpoint calculation (for example, preoperative maximal flexion <90°). The need for at least one blood transfusion will be compared between the study and the control group to demonstrate whether or not EVICEL™ reduces the need for transfusions. Furthermore, it should be shown that the use of analgesics (according to the WHO pain ladder [4]) can be reduced by treatment with EVICEL™ and that the time until the wound is completely dry is shorter. In addition, it should be shown that the clinical outcome in the EVICEL™ group is better than that in the control group. Clinical outcome is assessed by two different clinical outcome scores, the clinician-completed Knee Society Score (KSS) and the patient-completed Knee Injury and Osteoarthritis Outcome Score (KOOS) [5, 6].

For the overall cost of the treatment, differences in duration of hospital stay, as well as differences in the total cost of treatment between the two groups will be compared, including the need for physiotherapy. The length of the inpatient stay depends not only on the patient’s condition but also on social and organisational factors, such as the capacity of the inpatient rehabilitation centre. Therefore, a theoretical discharge date will be defined and evaluated in addition to the actual date. The theoretical discharge date is defined as the first day on which the patient achieves the following three criteria: a dry wound without signs of infection, maximum knee flexion of at least 90° and the ability to climb stairs using crutches.

For the sake of safety, patients will be under permanent surveillance during the operation. Adverse events (AEs) and serious adverse events (SAEs) will be documented. Clinical signs of infections will be monitored daily, and blood parameters collected regularly.

### Study population

A total of 68 men or nonpregnant women scheduled for primary unilateral TKR will be recruited and treated in the context of this RCT.

The inclusion criteria are as follows:

1. Men or women aged from 55 to 70 years on the day of the operation.
2. Patients with an indication for primary TKR on the basis of diagnosed osteoarthritis, arthritis or avascular necrosis.
3. Patients with intact medial and lateral collateral ligaments (to be diagnosed in the preoperative examination) who are planned preoperatively to receive a Stryker Triathlon CR or Triathlon PS system prosthesis.
4. Patients willing to participate in this RCT and who have signed the informed consent form approved by the ethics committee.

The exclusion criteria are as follows:

1. Patients participating in any other clinical trial within 30 days before inclusion or concurrent with this study.
2. Pregnant or nursing women.
3. Patients tested positive for HIV, hepatitis B and C.
4. Patients with documented or suspected hypersensitivity to any of the active substances or excipients of EVICEL™, or to bone cement or its components.
5. Patients with acute or suspected coagulation disorders and patients with preoperative blood parameters INR >1.4 or PTT >40 s.
6. Concomitant medication within 7 days before surgery with substances that affect haemostasis, except for low molecular weight heparins or alternatives in prophylactic dosage indicated for standard thrombosis prophylaxis.
7. Anaemic patients showing preoperative Hb levels <11 g/dl.
8. Patients with a deficit in extension >15° or with a maximal flexion <90°.
9. Highly obese people (BMI >35).
10. Patients suffering from neuromuscular or neurosensory diseases.
11. Patients with known active tumour disease or tumour-related diseases.
12. Patients suffering from any significant concurrent disease, illness or psychiatric disorder that would compromise their safety or compliance, or interfere with consent, study participation, follow-up or interpretation of the results.
13. Patients receiving a prosthesis different from the Stryker Triathlon CR or Triathlon PS system owing to intraoperative circumstances (for example, bone fractures or ligament insufficiencies).
14. Intraoperative deviation from the agreed haemostatic procedure (for example, use of the tourniquet deviating from the agreement, for example in case of surgery lasting >2 h). Patients with surgery lasting >2 h will not be randomised.

### Study procedure

On the day of admission eligible patients will be informed about the study protocol. If they agree to participate in the study and sign and date the informed consent form, the following procedure will apply (Table 1):

**Table 1:** Schedule of events

Procedures	Baseline (within 72 h prior to procedure)	Day of surgery	Postoperative stay in hospital	3 Months after surgery ± 2 weeks	6 Months after surgery ± 2 weeks	12 Months after surgery ± 2 weeks
Inclusion/exclusion	X	X				
Randomisation		X				
Informed consent	X					
Demographics	X					
Medical history	X					
Concomitant medications including use of analgesics or peripheral nerve block	X	X	X^d^	X	X	X
Physical examination (ROM)	X		X^d^	X	X	X
Laboratory tests (virology^a^, CBC^b^, clinical chemistry^c^)	X^a–c^		X^e,b,c^			
Coagulation parameters (INR, PTT)	X					
Treatment application		X				
Intraoperative data assessment		X				
Clinical outcome score assessment (KSS/KOOS)	X		X^f^	X	X	X
Surgical site assessment			X^d^	X	X	X
Wound healing assessment			X^d^			
Assessment of patient’s mobility on crutches (stairs)			X^d^			
Adverse events		X	X^d^	X	X	X
Assessment of physiotherapy treatments after rehabilitation				X	X	X
Calculation of overall costs of TKR						X

^a^Virology: Hep B/C and HIV screening

^b^CBC: Hb, leucocytes

^c^Clinical chemistry: CRP

^d^Assessment daily during the visits (except for Sundays as for the physical examination)

^e^Assessment on days 2, 4 and 6 (+/− 1 day) and on day 7

^f^Assessment on day 7

#### Preoperative examinations (baseline)

The preoperative examinations to establish baseline measures will include the following:

1. Orthopaedic examination of the lower extremity, including measurement of ROM, and confirmation of the medical indication.
2. Blood samples taken and tested according to in-house protocol. Relevant parameters for the study are: INR, PTT and complete blood count (CBC) without differential, including Hb and leucocytes. The amount of blood needed for this routine procedure is about 26.5 ml.
3. Subcutaneous injection of certoparin-sodium (for example, Mono Embolex™) at a dose of 3,000 IU/day performed from the preoperative evening onwards. An equivalent drug might be used as an alternative, depending on the guidelines (for example, no preoperative administration in the case of some oral products).

The following general data will be collected on the corresponding case report form (CRF): patient ID; gender and date of birth; height and weight; BMI; medical history, especially regarding thromboembolic events; smoking status; comorbidities and concomitant medications. In addition, the following specific data will be assessed: indication(s) for TKR, Hb level (g/dl), C-reactive protein (CRP) (mg/dl), leucocytes (1/μl), virology (hepatitis B/C and HIV screening), haemostatic parameters (INR and PTT), ROM (angle of maximum flexion), details of medications used as prophylaxis against thrombosis, and assessment of KSS and KOOS.

#### Details of the operation and of EVICEL™ administration

Investigators will perform the operation according to the established in-house standard and the guidelines of the prosthesis manufacturer. The operation will follow a very strict protocol in order to minimise adverse factors. The prosthesis implanted will be the Stryker Triathlon™ system. All components are always cemented with antibiotic-containing cement (Refobacine Palacos). The operation will be performed in a bloodless field: prior to the skin incision, the tourniquet is set to 250 mmHg. During the operation haemostasis will be performed conventionally by electrocoagulation.

For the study group, haemostasis is extended by the use of EVICEL™ according to the manufacturer’s guidelines and recommendations. The application will be performed by the surgeons themselves. The first application takes place immediately after lavage, before implanting the prosthesis: 2 ml of EVICEL™ are sprayed into the popliteal fossa, which cannot be approached subsequently. Where cancellous bone surfaces are bleeding after cementation of the prosthesis, EVICEL™ is sprayed on those areas. After hardening of the bone cement, functional tests and conventional haemostasis are performed as usual. The second administration of EVICEL™ is carried out on the meniscal and capsular blood vessels as well as in the superior recess (a total of 2 ml). A wait of 2 min without any action is necessary before starting the suture. After suturing the joint capsule, 1 ml of EVICEL™ is sprayed into the subcutaneous tissue. Suction or swabbing must be strictly avoided in the areas where EVICEL™ has been administered.

In case of complications occurring during the administration of EVICEL™ (for example, intraoperative thromboembolic events or allergic reactions), the application will be stopped immediately in the subject concerned.

For both groups, a sterile compression bandage is placed after skin suturing. The tourniquet is then removed.

In order to ensure better comparability of the results, no wound drainage system will be used in this study as the drained volume depends on the hardly reproducible position of the drainage *in situ*.

The tourniquet must be removed after 2 h at the latest. If the operating time from incision to suturing exceeds 2 h, the tourniquet is removed earlier in the procedure. In this situation, the patient will be withdrawn from the study.

The following specific data concerning the operation are collected: duration of surgery; type of anaesthesia (spinal or general); management of fluid balance; use of tourniquet (pressure in mmHg and time in minutes); amount of EVICEL™ used (ml); intraoperative complications; use of peripheral/nerve blocks; number of allogeneic and autologous blood units transfused; and documentation of autologous retransfusion of blood collected during surgery.

#### Postoperative procedure

The postoperative procedure is described as follows:

1. Prophylaxis against thrombosis is continued with certoparin-sodium 3,000 IU (or alternative) once daily until the patient reaches a physiological level of activity.
2. The bandages are removed on day 1 after surgery. Physiological exercises, manual lymphatic drainage and mobilisation of the patient under full weight-bearing begin on day 1, according to the established in-house standard. Sutures are removed between days 10 and 14.
3. The standard postoperative transfusion criterion at the site is an Hb level <6 g/dl, or in case of values between 6 and 8 g/dl, more than mild symptoms of anaemia. However, the criteria are soft and must be adapted according to the patient’s individual situation and his/her comorbidities, especially any cardiac comorbidities. The decision for or against a transfusion is therefore made by the treating anaesthetist or orthopaedic surgeon, according to their clinical experience. Thus, the unlikely possibility of transfusing patients with Hb levels between 8 and 10 g/dl cannot be completely ruled out. For each transfusion performed, the reasons will be documented precisely.
4. Collection of blood parameters, their analysis and the documentation of the secondary endpoints are carried out as described in point 6 (Assessment of Efficacy). The amount of blood taken for each of the four blood tests is about 8.2 ml.

The following postoperative data are collected in the corresponding CRF (parameters marked with an asterisk are also assessed during the follow-up examinations):

1. Blood parameters: Hb level 7 days postoperatively, CRP, leucocytes
2. In case of blood transfusions: number of units transfused, date of transfusion, Hb value before transfusion, reason for transfusion
3. ROM: angle of maximum flexion*
4. Use of analgesics (WHO pain ladder)*
5. Time when nerve block catheter is removed (if appropriate)
6. Complications (for example, disorders of wound healing, thromboembolic events, signs of infection, for example, abnormal redness, swelling, pain, loss of function, hyperthermia)*
7. Time when the wound is completely dry and any kind of secretion has ceased
8. Duration of hospital stay
9. KSS and KOOS assessments*
10. Type of rehabilitation after hospital stay (stationary, ambulant or other)*
11. Number of physiotherapy treatment units after rehabilitation, including manual lymphatic drainage*
12. Total costs of TKR
13. Theoretical date of discharge (defined in section ‘Primary and Secondary Study Endpoints’)

### Statistics

#### Sample size calculation

Statistical analysis of the published clinical evidence regarding the fibrin sealant QUIXIL, demonstrating its effects in controlling haemostasis and reducing postoperative blood loss after TKR, was used as a basis to calculate the number of patients needed for this RCT. Based on the literature [7], a sample size estimation for changes in Hb level was made with the statistical program nQuery Advisor 5.

The estimation assumes the following mean values and standard deviations (SD) of postoperative decreases in Hb [7]: 25 g/l (SD 10) (study group) and 37 g/l (SD 12) (control group). The level of significance is set at *P* = 0.05 and a two-sided *t*-test is performed with equal numbers from the two groups. With a power of 90 % and an estimated SD of 15 g/l, the required number of cases is 34 per group. For this sample size estimation we assumed that the use of additional information such as baseline Hb levels as covariates would increase the power of the test.

**Fig 1:**
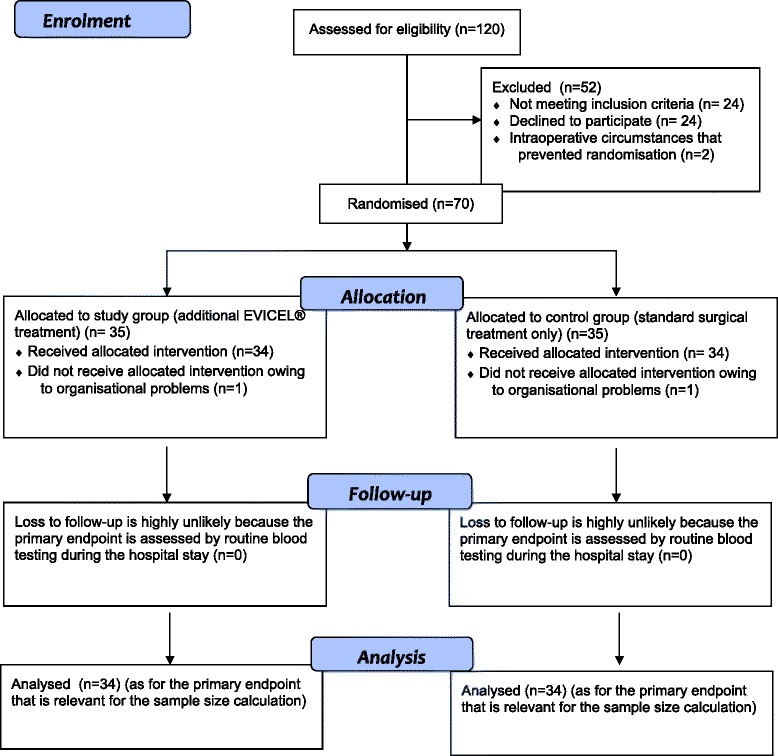
Flow diagram of study participants. The CONSORT flow diagram shows the anticipated number of patients from enrolment until analysis

For a total sample size of 68, approximately 120 patients must be screened because about 40 % of the eligible patients are expected either not to meet the inclusion criteria or not to consent to participate, owing to inconvenience with the follow-up examinations; not being randomised due to intraoperative circumstances; or to drop out for other reasons. Since the primary endpoint is blood loss during the first 7 days, which is calculated using routine blood tests during the inpatient stay, no drop-outs regarding the primary endpoint should be expected after surgery (Fig. 1).

#### Statistical methods

The statistical analysis will be carried out at the end of the study; interim analyses are not intended. However, the analysis of the primary endpoint may be carried out earlier, as soon as the data from the last patient have been assessed. For this analysis, ANCOVA for Hb level differences baseline with respect to the detected minimum in the first 7 postoperative days for both treatment groups with adjustment for baseline Hb levels will be used. If the upper limit of the two-sided 95 % confidence interval (CI) of the difference in means between EVICEL™ and standard orthopaedic surgery as estimated from the ANCOVA model is <0, the superiority of EVICEL™ will be concluded. Missing values for Hb observations will be replaced by the last observation carried forward method (LOCF). If there are no postoperative Hb values available for a particular patient, that patient will be recorded as having the largest decrease in Hb of the control group.

AEs and SAEs will be evaluated descriptively by chi-squared tests.

For the secondary endpoints, the need for at least one transfusion will be analysed by odds ratios (OR) together with 95 % CIs. The ROM and the length of hospital stay will be analysed by *t*-tests. The total cost per patient will be analysed using Wilcoxon’s signed ranks test. Time until wound healing will be tested by log rank tests.

Secondary and tertiary analyses are exploratory and will be performed descriptively.

The *P* values will be assessed descriptively and will be deemed significant when *P* <0.05.

The primary analysis will be conducted on the intention to treat (ITT) population, that is, for all randomised patients. Sensitivity analyses will be performed in the per-protocol (PP) population, including all patients who completed the study according to the protocol.

#### Randomisation/blinding

The process of randomisation is performed centrally by the Institute for Biometry. A randomisation list is created and assigns the patients to either the control group or the study group. When the surgical procedure arrives at the point at which EVICEL™ treatment has to be performed in the active group (after conventional haemostasis), the investigator then initiates the randomisation and queries the patient’s assignment at the Institute for Biometry by phone. This procedure allows the statement to be made that the operation prior to the application of EVICEL™ is concealed and performed without any bias.

In this RCT, the patients will be blinded but owing to the nature of the intervention the surgeons clearly cannot be blinded.

The treating surgeons will conduct the therapy in a way that complies with the single-blinded design of the study. In the rare case of a spinal anaesthesia, it will be ensured that patients will have no opportunity to realise whether they receive the medicinal product under investigation (IMP) or not. The medical team involved in the operation and patient’s care is responsible for keeping the treatment blinded. The nurses, physiotherapists and physicians collecting data after surgery will also be blinded to the treatment allocation.

### Subject withdrawal

As the application of EVICEL™ is conducted only at a single time point in a three-step procedure, subjects cannot be withdrawn from the IMP treatment. The only exceptions are complications occurring during the operation, leading to an immediate cessation of EVICEL™ use.

Patients undergoing a thromboembolic or any other event demanding anticoagulation that exceeds the standard prophylaxis measures during the first 7 days after surgery will be recorded as having the smallest Hb value before anticoagulation. Patients receiving anticoagulation during the operation will be recorded as having the largest Hb difference of the control group. The same procedure applies to patients receiving any kind of blood product during or after the operation. Because in nearly all cases there will be a blood sample collection prior to a transfusion or to a change in anticoagulation medication, these patients are expected to account for <1 % of all patients and will be recorded separately.

The following procedure applies to patients receiving any kind of blood product or an extended anticoagulation medication during or after the operation. Patients receiving blood transfusions during the operation but before randomisation will not be randomised. Patients receiving blood transfusions during the operation and after randomisation will be recorded as having the largest Hb difference of the control group. Patients receiving a blood transfusion after the operation would require measurement of Hb level before transfusion. Patients deteriorating despite transfusion will be recorded as having the smallest observed Hb level. For each transfusion, the Hb value at the time of transfusion, the reason for the transfusion (with specification of the symptoms) and the time of transfusion will be documented precisely.

Patients with an intraoperative deviation from the agreed haemostatic procedure (use of EVICEL™, electrocoagulation, or of the tourniquet deviating from the agreement, e.g. in the case of surgery lasting >2 h) will not be enrolled. In most cases, it is foreseeable before randomisation that the operation will probably take longer than 2 h; therefore, those patients will not be randomised, and the percentage of subjects not included because of the duration of surgery is expected to be <1 %.

### Insurance

Mandatory patient insurance for this trial according to AMG § 40 (3) has been obtained. Because of this, any damage to patient health during the conduct of the study will be insured, with a maximum amount of coverage of €500,000 per patient. This covers all damage that may occur to the patient either indirectly or directly as a result of the study medication or interventions in connection with the RCT.

### Overview of the medical product under investigation

EVICEL™ is a fibrin sealant kit consisting of two human plasma-derived components, human clottable protein containing mainly fibrinogen and fibronectin (component 1), and human thrombin (component 2), both produced by Omrix Biopharmaceuticals S.A. [8].

EVICEL™ is a further development of QUIXIL, which has been approved for marketing in 14 EU countries since 2003, first in the UK in 1999. One difference between EVICEL™ and QUIXIL is the final composition of the fibrinogen component, but the thrombin component remains the same. The fibrinogen component of QUIXIL contains the synthetic antifibrinolytic agent tranexamic acid (TA), which inhibits the degradation of fibrinogen. However, because TA is potentially neurotoxic QUIXIL is contraindicated for use in neurosurgery and all procedures where contact with the cerebral spinal fluid (CSF) and dura mater might occur. By specifically removing plasminogen the need for stabilisation with TA is avoided, and EVICEL’s fibrinogen is formulated without TA. Its protein concentration is 30 to 50 % higher, requiring the submission of a new application for marketing authorisation.

EVICEL is indicated as an adjunct to haemostasis for use in patients undergoing surgery, when control of bleeding by standard surgical techniques (such as sutures, ligatures or cautery) is ineffective or impractical. EVICEL™ is intended for epilesional use only, and the dosage should always be oriented towards the underlying clinical needs of the patient. The manufacturer recommends a dosage of 5 ml for TKR.

### Risks

The following potential risks may occur when administering fibrin sealants:

1. Immunogenicity, hypersensitivity, allergic reaction and anaphylaxis, and the possible development of antibodies against components of fibrin sealants. The risk is markedly increased when fibrin sealants are used repeatedly or in patients with a known hypersensitivity to the active substances or to any of the excipients.
2. Thromboembolic events due to accidental intravascular administration.
3. Transmission of infections. Standard measures to prevent infections resulting from the use of medicinal products prepared from human blood or plasma include the selection of donors, screening of individual donations and plasma pools for specific markers of infection, and the inclusion of effective manufacturing steps for the inactivation/removal of viruses. The measures taken are considered effective for enveloped viruses such as HIV, hepatitis C virus and hepatitis B virus, and for the non-enveloped hepatitis A virus. The measures taken may be of limited value against non-enveloped viruses such as parvovirus B19.
4. Complications related to infections or thromboembolism, postoperative wound complications.
5. Tissue adhesion at undesired sites owing to incorrect product application.

## Discussion

The clinical significance of and rationale for the conception of this study was the fact that after TKR only the ‘visible’ blood loss is usually known, whereas ‘hidden’ blood loss often is underestimated. In a study involving 101 patients undergoing TKR, the hidden blood loss contributed 49 % to the ‘true’ total blood loss from bleeding into the tissues or into the joint [1].

A decrease in postoperative Hb levels may be caused by either intraoperative or postoperative blood loss. EVICEL™ is used at the end of the surgery, so that postoperative blood loss is the only fraction that might be affected by its use. Therefore, the study was designed to keep intraoperative blood loss as low as possible in order to provide better conditions for the effect of the fibrin sealant and to achieve meaningful results. For this reason, all operations were planned to be conducted in a bloodless field.

Several clinical studies have shown that the preceding fibrin sealant, QUIXIL, significantly reduces blood loss in patients undergoing total hip or total knee replacement [7, 9–11]. In contrast, another study evaluating blood loss and number of blood transfusions in patients undergoing TKR could not prove any benefit from the use of QUIXIL [12]. In a study with 165 patients undergoing TKR, the authors concluded that the use of platelet gel and fibrin sealant improves ROM, reduces hospital stay and may reduce the incidence of arthrofibrosis [2]. However, during the planning phase of the study protocol presented here, there were no data concerning the newer fibrin sealant EVICEL.

Owing to delays in obtaining approval for the study from the competent national authority associated with product changes, by the time the study was finally ready for submission there were several published studies in the literature evaluating the effects of EVICEL in TKR. Randelli *et al*. [13] reported in their RCT that the application of EVICEL reduces neither perioperative blood loss nor the need for allogeneic blood transfusion. Skovgaard *et al*. [14] found that the drain output in knees treated with fibrin sealant and those treated with placebo was similar, and that no statistically significant differences could be seen regarding either swelling, pain, strength of knee extension or ROM.

In another study [15], however, the results suggested that transfusion rates in anaemic patients undergoing TKR were significantly lower than in a control group, and that the use of EVICEL resulted in a significant reduction of blood loss. In their meta-analysis, Liu *et al*. [16] reported that the use of fibrin sealants in TKR significantly reduced total blood loss, drainage blood loss and haemoglobin loss as well as transfusion rates.

In conclusion, a literature search delivers inconsistent and ambivalent results indicating that, despite the presence of comparable studies, there is still a need for high-evidence studies clarifying the role of fibrin sealants in TKR, as proposed in the study protocol presented here.

## Trial status

This RCT was designed as an investigator-initiated study for which Hannover Medical School was assumed to be acting as sponsor. The study was funded by Ethicon Inc., the parent company of Omrix Biopharmaceuticals, which produces the IMP. A contract was signed and initial financial support was provided according to the contract terms, which included approval by the Independent Ethics Committee. Approval by the national competent authority was not obtained following feedback from the European Medicines Agency. When the issues were resolved, the study was prepared for resubmission to the national competent authority. However, in the meantime, ETHICON changed their business strategy and further funding was withdrawn. Therefore, the study has not yet started patient recruitment.

## Abbreviations

AE: adverse event
AMG: Arzneimittelgesetz (German Drug Law)
ANCOVA: analysis of covariance
BMI: body mass index
CBC: complete blood count
CRF: case report form
CRP: C-reactive protein (blood marker of infection)
CSF: cerebral spinal fluid
DIC: disseminated intravascular coagulation
DRKS: Deutsches Register Klinischer Studien (German Registry of Clinical Trials)
Hb: haemoglobin
HIV: human immunodeficiency virus
ID: identification
IMP: investigational medicinal product
INR: International Normalised Ratio
ITT: intention to treat
KSS: Knee Society Score
KOOS: Knee Injury and Osteoarthritis Outcome Score
LOCF: last observation carried forward
OR: odds ratio
PP: per protocol
PTT: partial thromboplastin time
RCT: randomised controlled trial
ROM: range of motion
SAE: serious adverse event
TKR: total knee replacement
TA: tranexamic acid

## References

[1] Sehat KR, Evans RL, Newman JH (2004). Hidden blood loss following hip and knee arthroplasty. Correct management of blood loss should take hidden loss into account. J Bone Joint Surg Br.

[2] Everts PAM, Devilee RJ, Oosterbos CJ, Mahoney CB, Schattenkerk ME, Knape JT (2007). Autologous platelet gel and fibrin sealant enhance the efficacy of total knee arthroplasty: improved ROM, decreased length of stay and a reduced incidence of arthrofibrosis. Knee Surg Sports Traumatol Arthrose..

[3] Gogarten W, Van Aken H (2009). Die neue S3-Leitlinie zur Thromboembolieprophylaxe-Bedeutung für unser Fachgebiet. Anästh Intensivmed..

[4] Ventafridda V, Saita L, Ripamonti C, De Conno F (1985). WHO guidelines for the use of analgesics in cancer pain. Int J Tissue React..

[5] Insall JN, Dorr LD, Scott RD, Scott WN (1989). Rationale of the Knee Society clinical rating system. Clin Orthop Relat Res..

[6] Roos EM, Roos HP, Lohmander LS, Ekdahl C, Beynnon BD (1998). Knee injury and osteoarthritis outcome score (KOOS) - development of a self-administered outcome measure. J Orthop Sports Phys Ther..

[7] Levy O, Martinowitz U, Oran A, Tauber C, Horoszowski H (1999). The use of fibrin tissue adhesive to reduce blood loss and the need for blood transfusion after total knee arthroplasty. J Bone Joint Surg..

[8] Assessment Report for Evicel. European Medicines Agency. Doc.Ref.: EMEA/CHMP/449199/2008. http://www.ema.europa.eu/docs/en_GB/document_library/EPAR_-_Public_assessment_report/human/000898/WC500030826.pdf

[9] Wang GJ, Hungerford DS, Savory CG, Rosenberg AG, Mont MA, Burks SG (2001). Use of fibrin sealant to reduce bloody drainage and hemoglobin loss after total knee arthroplasty: a brief note on a randomized prospective trial. J Bone Joint Surg Am..

[10] Wang GJ, Goldthwaite CA, Burks S, Crawford R, Spotnitz WD, Orthopaedic Investigators Group. (2003). Fibrin sealant reduces perioperative blood loss in total hip replacement. J Long Term Effects Med Implants..

[11] Crawford RW, Giangrande P, Murray D (1999). Fibrin sealant reduces blood loss in total hip arthroplasty. Hip Int Off J European Hip Soc..

[12] Kluba T, Fiedler K, Kunze B, Ipach I, Suckel A (2012). Fibrin sealants in orthopaedic surgery: practical experiences derived from use of QUIXIL® in total knee arthroplasty. Arch Orthop Trauma Surg..

[13] Randelli F, D’Anchise R, Ragone V, Serrao L, Cabitza P, Randelli P (2014). Is the newest fibrin sealant an effective strategy to reduce blood loss after total knee arthroplasty? A randomized controlled study. J Arthroplasty..

[14] Skovgaard C, Holm B, Troelsen A, Lunn TH, Gaarn-Larsen L, Kehlet H (2013). No effect of fibrin sealant on drain output or functional recovery following simultaneous bilateral total knee arthroplasty: a randomized, double-blind, placebo-controlled study. Acta Orthop..

[15] Bou Monsef J, Buckup J, Waldstein W, Cornell C, Boettner F (2014). Fibrin sealants or cell saver eliminate the need for autologous blood donation in anemic patients undergoing primary total knee arthroplasty. Arch Orthop Trauma Surg..

[16] Liu J, Cao JG, Wang L, Ma XL (2014). Effect of fibrin sealant on blood loss following total knee arthroplasty: a systematic review and meta-analysis. Int J Surg..

